# Establishment of a CRISPR/dCas9 Activation Library for Screening Transcription Factors Co-Regulating OCT4 with GATA4 in Pig Cells

**DOI:** 10.3390/cells14171330

**Published:** 2025-08-28

**Authors:** Xiaoxia Yao, Mingjie Feng, Chengbo Sun, Sijia Yang, Zhongyu Yuan, Xueqing Liu, Qinjian Li, Chaoqian Jiang, Xiaogang Weng, Jun Song, Yanshuang Mu

**Affiliations:** 1 Key Laboratory of Animal Cellular and Genetic Engineering of Heilongjiang Province, Northeast Agricultural University, Harbin 150038, China; 2 College of Life Science, Northeast Agricultural University, Harbin 150038, China

**Keywords:** pig, CRISPR sgRNA library, *OCT4*, *GATA4*, transcriptional regulation

## Abstract

*OCT4* is a critical transcription factor for early embryonic development and pluripotency. Previous studies have shown that the regulation of *OCT4* by the transcription factor *GATA4* is species-specific in pigs. This study aimed to further investigate whether there are other transcription factors that co-regulate the transcription of *OCT4* with *GATA4* in pigs. A CRISPR activation (CRISPRa) sgRNA library was designed and constructed, containing 5056 sgRNAs targeting the promoter region of 1264 transcription factors in pigs. Then, a pig PK15 cell line was engineered with a single-copy *OCT4* promoter-driven EGFP reporter at the ROSA26 locus, combined with the dCas9-SAM system for transcriptional activation. The CRISPRa sgRNA lentiviral library was used to screen for transcription factors, with or without *GATA4* overexpression. Flow cytometry combined with high-throughput sequencing identified *MYC*, *SOX2*, and *PRDM14* as activators and *OTX2* and *CDX2* as repressors of *OCT4*. In the presence of *GATA4*, transcription factors such as *SALL4* and *STAT3* showed synergistic activation. Functional validation confirmed that *HOXD13* upregulates *OCT4*, while *OTX2* inhibits it. *GATA4* and *SALL4* synergistically enhance *OCT4* expression. These findings provide new insights into combinatorial mechanisms that control the transcriptional regulation of *OCT4* in pigs.

## 1. Introduction

Pig embryos are of fundamental importance to the pig industry, as they contribute significantly to improving reproductive efficiency, accelerating genetic progress, maintaining herd health, and increasing economic returns [1,2]. To address key challenges such as early embryonic loss and suboptimal reproductive performance in sows, it is essential to elucidate the molecular mechanisms governing early embryonic development and lineage differentiation [1,3]. A central regulator in these processes is *OCT4* (octamer-binding transcription factor 4), a core transcription factor that maintains the pluripotency and self-renewal capacity of embryonic stem cells (ESCs) [4]. The expression level of *OCT4* serves as a critical determinant of a cell’s fate: low expression favors self-renewal, moderate expression sustains pluripotency, and high expression induces differentiation [5,6,7]. During embryogenesis, OCT4 plays a key role in directing the differentiation of ectodermal and endodermal lineages [8]. Its absence leads to the differentiation of ESCs into trophoblast cells and a loss of pluripotency within the inner cell mass (ICM) [9]. Moreover, mutations in *OCT4* impair blastocyst formation and prevent ESCs from maintaining ectoderm-like characteristics [8]. These findings highlight the necessity of the precise regulation of *OCT4* expression to ensure normal embryonic development.

Comparative sequence analysis has revealed a high degree of evolutionary conservation of the OCT4 protein among mammals. Despite this conservation, species-specific differences in the upstream regulatory region of *OCT4* contribute to distinct regulatory mechanisms. The upstream region contains four conserved regions (CR1–CR4), among which CR4 exhibits the highest interspecies variability and harbors several critical transcription factor binding sites, including Site2A, Site2B (OCT4/SOX2 sites), and the AP2 site [10]. In non-rodent species, CR4 additionally contains a unique oligonucleotide motif, AGAT/G (N1 site), which forms a canonical GATA-binding site essential for *OCT4* promoter activity in trophectoderm (TE) cells [11,12]. This motif is conserved in pigs, cattle, sheep, and rabbits but is absent in rodents such as mice and rats, explaining why *GATA4* can activate *OCT4* expression in TE cells of non-rodent species but not in mice [11]. Moreover, while SOX2-OCT4 heterodimers can bind conserved motifs to promote *OCT4* expression [10,13,14], the absence of *SOX2* in murine TE cells leads to silencing of *OCT4* in that lineage [15]. In contrast, *GATA4* in non-rodent species compensates for early *SOX2* downregulation by sustaining *OCT4* expression in TE cells [11]. In pigs, an additional regulatory layer is observed in the interaction between *OCT4* and *CDX2*, where *CDX2* overexpression does not alter *OCT4* transcription or translation but inhibits its DNA-binding activity through competitive interference, promoting OCT4 degradation and nuclear export [11]. These findings collectively reveal that although *OCT4* is highly conserved among mammals, its transcriptional regulation in TE cells displays species-specific features, particularly in non-rodent animals like pigs, where *GATA4* plays a crucial compensatory role in maintaining *OCT4* expression during early embryogenesis.

The CRISPR activation (CRISPRa) system enables the transcriptional activation of endogenous genes by targeting specific promoter or enhancer regions using a catalytically inactive Cas9 (dCas9) fused to transcriptional activation domains—such as VP64 or the p300 core domain—guided by sequence-specific single guide RNAs (sgRNAs) [16]. This system initiates transcription by altering chromatin conformation or recruiting transcriptional and epigenetic co-activators to target loci [17,18]. To improve activation efficiency, various CRISPRa platforms have been developed, including the dCas9–VP64 system [19], dCas9–VPR [20], sgRNA scaffold-based systems such as dCas9–MCP [21], the synergistic activation mediator (SAM) system [22], SunTag [17,23], and newer systems like dCas9–VPRP, TREE, and enCRISPRa [24,25,26]. These systems have been successfully applied across species to achieve targeted gene activation [27]. In pigs, the dCas9-SAM system has shown a robust activation of endogenous genes in PK15 and IPEC-J2 cell lines [28]. To facilitate large-scale transcriptional regulation studies and functional genomics in pig models, we established a CRISPR-based lentiviral activation library for transcription factor screening, enabling high-throughput gain-of-function (GOF) screening and the systematic identification of key transcription factors involved in gene expression and developmental pathways in pigs [29].

In this study, a CRISPRa screening approach was used to identify transcription factors that regulate *OCT4* expression and potentially act together with *GATA4* in pigs. A pig’s sgRNA library targeting the promoter regions of 1264 transcription factors was constructed. Using a PK15 reporter cell line with an *OCT4* promoter-driven EGFP inserted at the ROSA26 locus and co-expressing the dCas9-SAM system, we performed high-throughput screening with or without *GATA4* overexpression. Flow cytometry and sequencing revealed that *MYC*, *SOX2*, and *PRDM14* activate *OCT4*, while *OTX2* and *CDX2* repress it. In the presence of *GATA4* overexpression, *SALL4* and *STAT3* show synergistic effects. Further validation confirmed that *HOXD13* enhances *OCT4* expression, while *OTX2* suppresses it. *GATA4* and *SALL4* act cooperatively to promote *OCT4* transcription. This study provides a new perspective on the transcriptional regulation mechanism of pigs’ *OCT4* by *GATA4*.

## 2. Materials and Methods

### 2.1. Cell Culture

PK15 pig kidney cells were cultured in DMEM (Gibco, Grand Island, NY, USA) containing 15% fetal bovine serum (Gibco, Grand Island, NY, USA) at 37 °C in 5% CO_2_ in a humidified incubator.

### 2.2. Plasmid Construction

#### 2.2.1. Construction of pOCT4-EGFP-TK Knock-In Plasmid

The TK fragment was amplified by PCR (See Supplementary Table S2-1, Primers for amplification of the TK fragment) and cloned into a BsmBI-linearized pOCT4-EGFP vector using the T4 DNA ligase (New England Biolabs, Ipswich, MA, USA). The ligation mixture was transformed into *E. coli*, and positive clones were screened by colony PCR, confirmed by Sanger sequencing, and validated via restriction digestion. The verified plasmid was extracted using a plasmid miniprep kit (Takara, Dalian, China), linearized with SgrDI (ThermoFisher, Shanghai, China), purified, and stored for subsequent use.

#### 2.2.2. Construction of pLenti 4×tRNA-sgGATA4 Plasmid

sgRNAs targeting *GATA4* were designed using the CRISPOR tool (http://crispor.tefor.net/). Tandem tRNA–sgRNA sequences (Supplementary Table S3) were synthesized (GenScript, Nanjing, China) and cloned into the pLenti_sgRNA(MS2)_Zeo backbone vector. The recombinant plasmid was transformed into competent cells, and colonies were validated by Sanger sequencing and restriction digestion. Verified clones were amplified and stored at −20 °C.

#### 2.2.3. Validation of sgRNA Expression Vectors

The CRISPRa-Common plasmid was linearized by BsmBI (New England Biolabs, Ipswich, MA, USA) digestion at 55 °C for 12 h before being purified. Single-stranded sgRNA oligos were annealed by heating to 95 °C for 5 min and slowly cooled to 25 °C (0.1 °C/s). The annealed sgRNA duplexes were ligated into the digested vector using the T4 DNA ligase. The ligation products were transformed into competent cells, and colonies were selected for sequencing and plasmid amplification.

### 2.3. Establishment and Verification of EGFP Reporter Cell Line

PK15 cells were electroporated with the Cas9 protein, sgRNA, and a donor plasmid using optimized parameters. Using a CUY21EDIT II electroporator (BEX Co., Ltd., Tokyo, Japan), the electroporation program was run with the following parameters: a voltage of 200 V, a duration of 1 ms, and 5 pulses. After 24 h, the medium was changed, and G418 (Absin, Shanghai, China) selection was initiated on day 5. Positive clones were isolated by limiting dilution and culturing in 96-well plates. Single colonies were expanded and verified by PCR following cell lysis with RIPA buffer and Proteinase K (Takara, Dalian, China). The knock-in efficiency was assessed by PCR amplification and 1% agarose gel electrophoresis. See Supplementary Table S2-2 for Gene knock-in identification primers.

### 2.4. Establishment of GATA4 Activating Expression Cell Lines

The pLenti 4×tRNA-sgGATA4 construct was packaged as a lentivirus, and target cells were infected 24 h after seeding at approximately 50% confluency using antibiotic-free medium supplemented with 0–8 μL of viral supernatant. After 48 h, cells were harvested for RNA extraction using TRIzol reagent (Takara, Dalian, China), followed by chloroform extraction and isopropanol precipitation. Total RNA was reverse-transcribed using a standard reverse transcription system, and the *GATA4* expression levels were quantified by RT-qPCR. See Supplementary Table S2-3 for Q-PCR primers.

### 2.5. sgRNA Library Infection

The Pig Transcription Factor sgRNA Library contained a total of 5056 sgRNAs, targeting 1264 transcription factors. Oligos of the sgRNA library were synthesized via a programmable microarray and cloned as a pool into the pLenti_sgRNA (MS2) vector (GenScript, Nanjing, China). Library quality was assessed by NGS with a coverage of 100% (GenScript, Nanjing, China). The packaging of the sgRNA lentiviral library was completed by the Nanjing Genscript company.

Pig cells were cultured at 37 °C with 5% CO_2_ and infected using the lentiviral sgRNA library at an MOI of 0.3 to ensure that each cell received only one sgRNA. After 24 h, the medium was replaced, and cells were further cultured for another 24 h. Then, bleomycin (Sigma, Shanghai, China) selection was applied until all the cells in the control group had died, after which flow cytometry (BD, East Rutherford, NJ, USA) was performed.

Genomic DNA was extracted from flow cytometry-sorted cells using a genomic DNA extraction kit (Takara, Dalian, China). sgRNA regions were amplified by PCR using the following specific primers: screen-sgRNA-F (5′-GGACTATCATATGCTTACCGTAACTTG-3′) and screen-sgRNA-R (5′-CCAAGTTGATAACGGACTAGCCTTAT-3′). The PCR products were subjected to deep sequencing (Kaitai Biotechnology, Hangzhou, China).

### 2.6. Validation of Candidate Transcription Factors at the Transcriptional Level

To validate candidate transcription factors at the transcriptional level, CRISPR activation plasmids were electroporated into cells. The plasmid mixture was electroporated according to a preset program (the same as for PK15 cells), and cells were cultured for 48 h before puromycin (Sigma, Shanghai, China) selection. After drug selection, cells were harvested for RNA extraction, reverse transcription, and RT-qPCR to quantify OCT4 expression. The experiment is repeated three times, with three samples each time. See Supplementary Table S2-3 for Q-PCR primers.

### 2.7. Data Analysis

CRISPR screening data were analyzed using MAGeCK (Broad Institute, version 0.5.9, Cambridge, MA, USA). Initially, the mageck count command was employed to align sgRNAs from the FASTQ files to the reference library, generating a count matrix with default quality control settings (Phred ≥ 30) and normalization method (median-ratio). Subsequently, the mageck test command was invoked, utilizing the MAGeCK-RRA algorithm to identify significantly enriched transcription factors between different fluorescence levels and the unsorted group, with significance thresholds set at FDR < 0.05 and |log_2_ fold-change| ≥ 1. Downstream analysis was conducted using MAGeCK-Flute (Broad Institute, Version3.2.1, Cambridge, MA, USA), which included the log transformation of FDR values (−log_10_(FDR)) and the selection of genes with effect sizes greater than zero. Visualization was achieved through R-generated scatter plots and log_2_ ratio plots, and transcription factor enrichment was ranked by integrating results from three replicates.

Data were analyzed and plotted in GraphPad Prism 8 (GraphPad Software, Version 8.0.2, San Diego, CA, USA), with error bars representing mean ± SEM. For group comparisons, one-way ANOVA followed by Tukey’s honestly significant difference (HSD) post hoc test was applied to adjust for multiple comparisons across all experimental groups, with adjusted * *p* < 0.05 considered statistically significant. Each experiment included three replicates.

## 3. Results

### 3.1. Construction of Pigs Transcription Factor sgRNA Library and Pigs Cell Line Expressing pOCT4-EGFP and dCas9 SAM System

**Figure 1:**
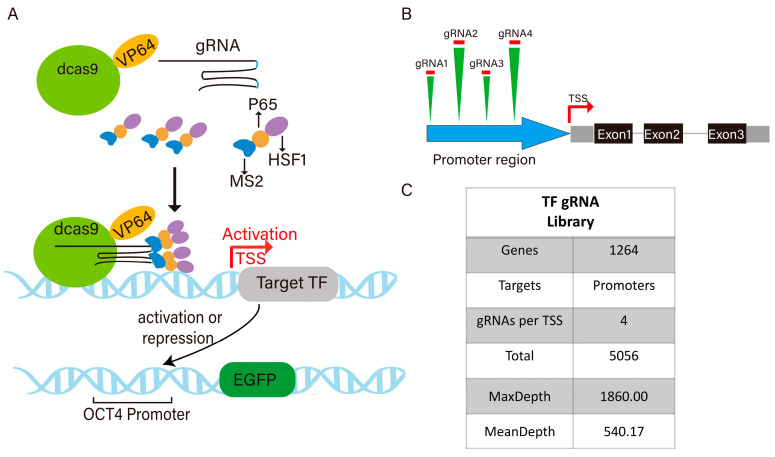
The principle of the SAM system activation and the overview of the sgRNA library. (**A**) Construction of pig transcription factor sgRNA library. (**B**) sgRNA design for pig transcription factor promoters. (**C**) Composition of sgRNA library.

To investigate the transcriptional regulatory network of *OCT4*, we utilized the established fluorescent reporter cell line expressing EGFP under the control of the *OCT4* promoter. Using the CRISPRa SAM system, where dCas9-VP64 is guided to gene promoters and further recruits p65 and HSF1 via MS2 RNA loops, we conducted high-throughput screening with a pig transcription factor (TF) CRISPRa library. Changes in EGFP fluorescence intensity served as a readout for *OCT4* activation or repression: increased fluorescence indicated TF-mediated activation, while a decreased signal suggested repression (Figure 1A). A previously constructed sgRNA library targeting 1264 pigs’ transcription factors (TFs) was used for CRISPRa-based screening. The library contains 5056 sgRNAs carrying MS2 loops, which were cloned into the lentiviral vector pLenti_sgRNA (MS2). The NGS results showed that the distribution of the sgRNA library was relatively uniform, with a maximum depth of 1860, an average depth of 540.17, and a coverage rate of 100%. Meanwhile, the library packaged by the lentivirus exhibited a high titer of 2.51 × 10^8^ IFU/mL, which ensured the effectiveness of the library in cell infection experiments (Figure 1B,C).

**Figure 2:**
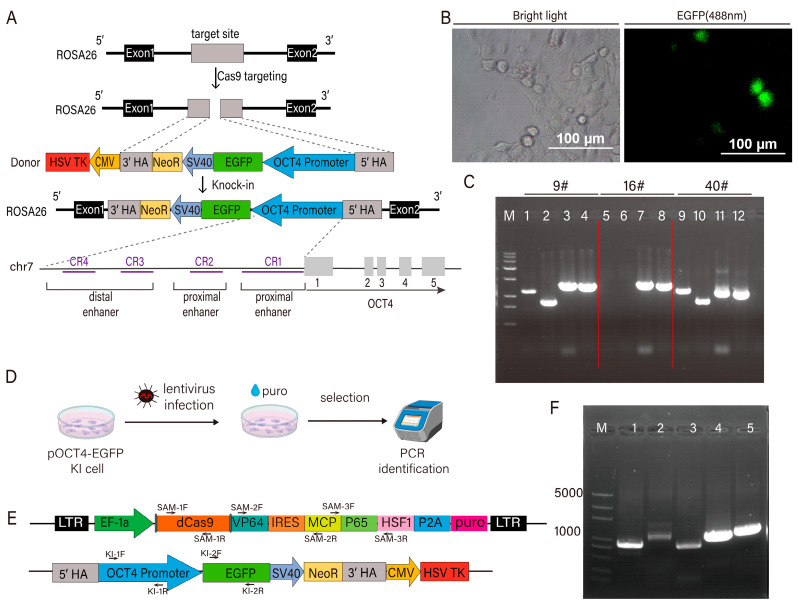
Construction of pig cell line expressing pOCT4-EGFP and dCas9 SAM system. (**A**) Targeted integration strategy for pig ROSA26 locus. (**B**) Knock-in identification microscopy image of pOCT4-EGFP. (**C**) PCR product electrophoresis image. Lane M is the marker; lanes 1–4 are the results for single cell clone # 9, lanes 5–8 are the results for single cell clone # 16, and lanes 9–12 are the results for single cell clone # 40. Lanes 3, 7, and 11 display the PCR results for the 5′ junction, with the expected knock-in fragment size of 1778 bp. Lanes 4, 8, and 12 correspond to the 3′ junction PCR, yielding a knock-in fragment of 1591 bp. Lanes 1, 9 and lanes 2, 10 represent the internal amplicons, with expected knock-in fragment sizes of 1430 bp and 1030 bp, respectively. (**D**) Lentiviral infection procedure. (**E**) Above: Schematic diagram of expression vector structure and PCR amplification target area of dCas9 SAM system; below: schematic diagram of pOCT4 EGFP-integrated PCR amplification target area. (**F**) PCR product electrophoresis image. Lane M is the marker; lanes 1–3 are the amplification products of the dCas9 SAM system components; lanes 4 and 5 are the amplified fragments of the pOCT4 EGFP target sequence.

The ROSA26 locus, first identified in mice, is widely used as a “safe harbor” site for gene knock-in due to its non-coding nature and minimal disruption to endogenous gene expression. In this study, a donor plasmid containing a 3.2 kb upstream regulatory sequence of the *OCT4* promoter-encompassing distal and proximal enhancers and the core promoter—was used to drive EGFP expression. To enable site-specific and homozygous integration, a negative selection marker (TK) was inserted between the homologous arms, complementing the Neo-positive selection system (Figure 2A). The expression of green fluorescence, PCR, and sequencing validation demonstrated that clone #9 is a homozygous knock-in of the pOCT4-EGFP cassette, confirming successful integration of the donor fragment (Figure 2B,C). Building on the successful generation of the pOCT4-EGFP knock-in PK15 cell line, we next introduced the SAM transcriptional activation system by integrating the lentiviral vector pLenti-dCas9. Lentiviruses were packaged in HEK293T cells and used to infect the knock-in PK15 cells. After 48 h, puromycin selection was applied for 15 days to establish stable cell lines co-expressing the OCT4-driven EGFP reporter and dCas9-SAM components (Figure 2D). Genomic DNA was extracted from the selected cells and analyzed by PCR and sequencing to confirm vector integration (Figure 2E,F).

### 3.2. Acquisition of GATA4-Activated Cells and Screening with sgRNA Library

**Figure 3:**
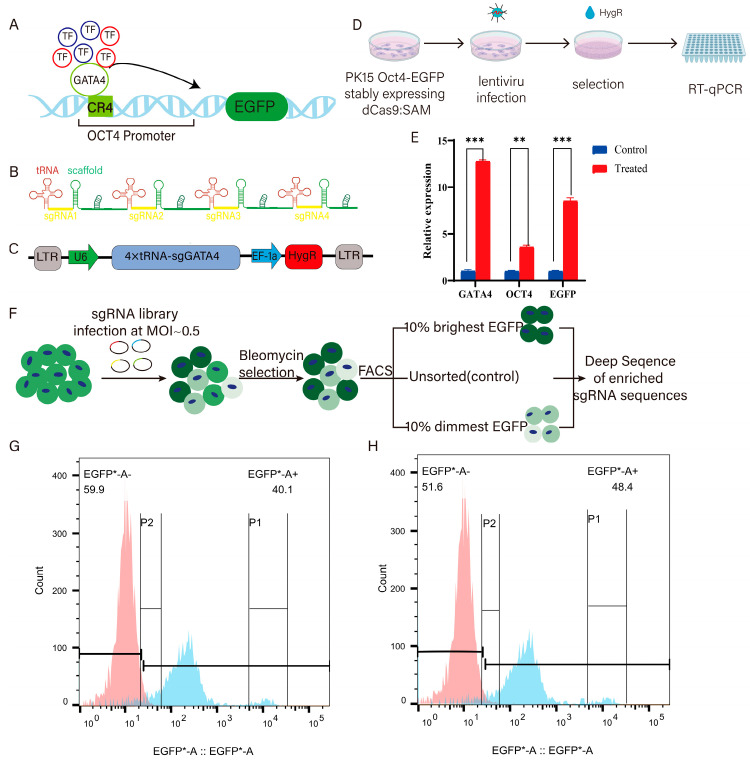
The acquisition and validation of cells with upregulated *GATA4* expression. (**A**) The schematic diagram of GATA4 cooperates with other transcription factors to regulate *OCT4*. (**B**) A schematic diagram of the tandem sgRNA structure. (**C**) A schematic diagram of the sequence structure of tandem sgRNAs. (**D**) A cell culture flowchart. (**E**) RT qPCR was used to detect the expression of *GATA4*, *OCT4*, and *EGFP* after viral infection. Note: * in the figure represents the significant difference between the two groups, where *** represents *p* < 0.001 and ** represents *p* < 0.01. (**F**) The screening process of the sgRNA library. (**G**) The flow cytometry gating strategy based on pOCT4-EGFP fluorescence intensity for cells expressing the dCas9 SAM system and pOCT4 EGFP knock-in after treatment with the sgRNA library (P1: EGFP + top 10%; P2: EGFP + bottom 10%). (**H**) The flow cytometry results of GATA4-activated cells after the same treatment (with the same gating criteria as **G**).

In pigs’ early trophoblast (TE) cells, the active expression of *OCT4* is highly dependent on the N1 site within its CR4 region, suggesting a critical role for *GATA4* in this regulatory process (Figure 3A). To explore potential co-regulatory transcription factors, we constructed an activation vector containing tandem sgRNAs targeting the *GATA4* promoter and introduced it into PK15 cells stably expressing both the pOCT4-EGFP reporter and the dCas9-SAM system. Promoter-targeting sgRNAs were designed within the −3000 bp to +50 bp region relative to the transcription start site (TSS), encompassing the regulatory and core promoter domains. To enhance activation efficiency, a tRNA–sgRNA array strategy was employed, leveraging tRNA-mediated cleavage for multiplexed sgRNA expression (Figure 3B,C). The resulting pLenti-4×tRNA-sgGATA4 vector was packaged into a lentivirus and used to infect the dual-expressing PK15 cells, followed by hygromycin selection and RT-qPCR analysis. The results showed a ~12-fold increase in *GATA4* expression, accompanied by a 3.5-fold and 8-fold upregulation of *OCT4* and *EGFP*, respectively, confirming the successful establishment of a GATA4-activated fluorescent reporter cell line (Figure 3D,E).

To identify the co-regulators of *OCT4* in coordination with *GATA4*, we compared screening results from two cell models: (1) pOCT4-EGFP knock-in cells expressing dCas9-SAM and (2) the same cells with enforced *GATA4* activation. By comparing the transcription factors identified in both conditions, we were able to distinguish those that uniquely synergized with *GATA4* in regulating *OCT4* expression (Figure 3F). To identify TFs directly regulating *OCT4* and those cooperating with *GATA4*, the sgRNA library was introduced into pOCT4-EGFP knock-in PK15 cells expressing the dCas9-SAM system. Infection was performed at MOI 0.5, followed by Bleomycin selection until the control cells were eliminated. Subsequently, cells with the top 10% and bottom 10% EGFP fluorescence were isolated via FACS (Figure 3G,H). A portion of unsorted cells was preserved as the “Unsort” group for baseline comparison. Cell counts were recorded to allow for a proportional analysis of sgRNA enrichment across sorted populations, facilitating the identification of candidate TFs modulating *OCT4* expression.

### 3.3. Data Analysis and Identification of Co-Regulated Genes

**Figure 4:**
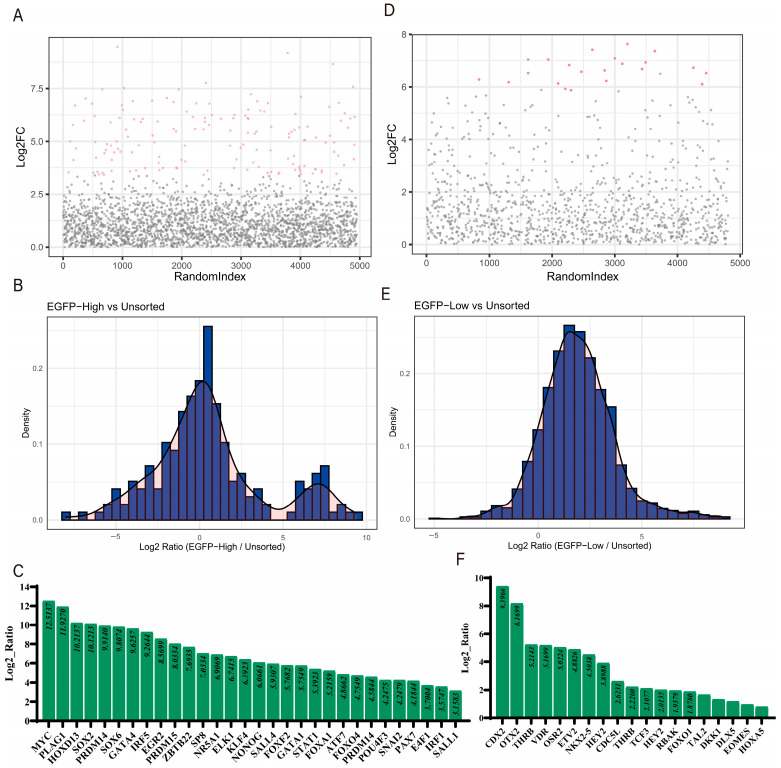
The enrichment analysis of transcription factors directly regulating *OCT4*. (**A**,**D**) Scatter plot: The horizontal axis represents the random index of genes, and the vertical axis represents the log2-fold change (Log2FC) of the genes, which is the expression difference between the experimental group and the control group. In the scatter plot, pink dots indicate genes with significant expression changes between the experimental group and the control group. (**B**,**E**) The log2 ratio distribution map shows the distribution of log2 ratio changes in gene expression between the experimental group (EGFP high/low) and the control group (unsorted). (**C**,**F**) The bar chart lists the top enriched genes in the experimental group, sorted from high to low according to their log2 ratio.

In the screening experiments following the library infection of cells expressing the dCas9-SAM system with pOCT4-EGFP knock-in, we identified significantly enriched genes in the EGFP-high cell population (Figure 4A), with higher Log2FC values indicating greater enrichment. Most gene expression ratios clustered around a log2 ratio of 0, suggesting minimal expression changes between the experimental group and the unsorted cell population (Figure 4B). However, certain genes, including *MYC*, *PLAG1*, *HOXD13*, *SOX2*, and *PRDM14*, were significantly enriched in this cell population (Figure 4C) and may potentially activate *OCT4* expression. In contrast, the EGFP-low cell population contained fewer but more concentrated enriched genes (Figure 4D), with most genes showing little expression change (Figure 4E). Genes with higher enrichment (Log2 ratio > 1), such as *CDX2*, *OTX2*, *THRB*, *VDR*, and *OSR2* (Figure 4F), may potentially repress *OCT4* expression (Supplementary Table S1).

**Figure 5:**
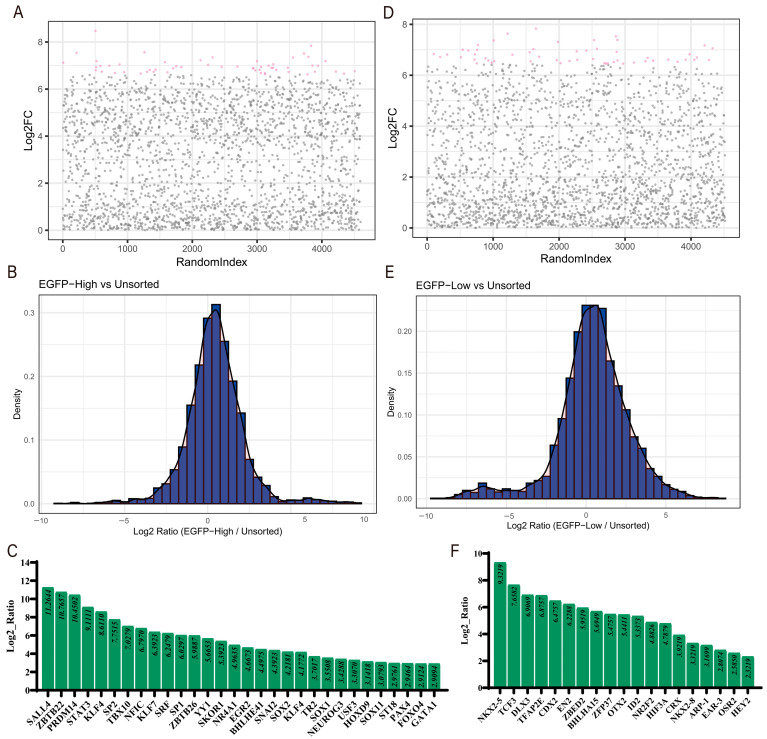
The enrichment analysis of transcription factors regulating *OCT4* through synergistic GATA4 regulation. (**A**,**D**) Scatter plot: The horizontal axis represents the random index of genes, and the vertical axis represents the log2-fold change (Log2FC) of genes, which is the expression difference between the experimental group and the control group. In the scatter plot, pink dots indicate genes with significant expression changes between the experimental group and the control group. (**B**,**E**) The log2 ratio distribution map shows the distribution of log2 ratio changes in gene expression between the experimental group (EGFP high/low) and the control group (unsorted). (**C**,**F**) The bar chart lists the top enriched genes in the experimental group, sorted from high to low according to their log2 ratio.

In the screening experiments following the infection of the fluorescent reporter cell population with activated *GATA4* expression and an integrated dCas9-SAM system, we analyzed the gene expression differences between the EGFP-high cell population and the unsorted cell population (Figure 5A). The distribution of gene expression ratios revealed that most genes had log2 ratios close to 0, while some genes were significantly enriched within the log2 ratio range of 5–10 (Figure 5B). The top 30 significantly enriched genes included *SALL4*, *ZBTB22*, *PRDM14*, *STAT3*, and *KLF4* (Figure 5C), which may potentially collaborate with *GATA4* to activate *OCT4* expression. In the screening of the EGFP-low cell population, the analysis of gene expression differences indicated that most relatively enriched genes had log2 ratios between 0 and 5 (Figure 5D,E). Significantly enriched genes such as *NKX2-5*, *TCF3*, *DLX3*, *TFAP2E*, and *CDX2* (Figure 5F) may potentially repress *OCT4* expression (Supplementary Table S1).

**Figure 6:**
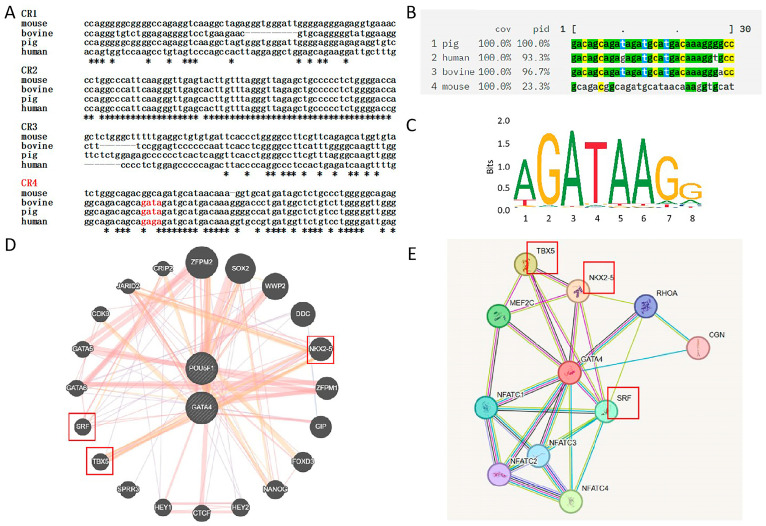
A multi-species *OCT4* promoter sequence alignment and conservation analysis. (**A**) The partial sequence alignment of the CR1-CR4 regions in the *OCT4* promoter across multiple species. (**B**) A statistics table of sequence conservation in the CR4 region of the *OCT4* promoter across multiple species. (**C**) The GATA-binding motif. (**D**) An interaction network diagram of *GATA4* and *OCT4*. (**E**) The protein–protein interaction network of *GATA4*. Note: In Figure A, the asterisks indicate that the nucleotides at these positions are identical in the corresponding gene regions across different species (mouse, cow, pig, and human). The transcription factors within the red boxes in Figures D and E indicate potential synergistic regulatory interactions with *GATA4*.

To investigate the conservation of the pigs’ *OCT4* promoter and explore GATA4-mediated regulation, Clustal Omega (Version 1.0) was used to perform a multiple sequence alignment of *OCT4* promoter regions from pigs, humans, bovines, and mice. The analysis focused on the CR4 region, revealing conserved GATA-binding sites in pigs, humans, and cattle, suggesting a potential regulatory role for *GATA* transcription factors in *OCT4* expression (Figure 6A–C). To thoroughly investigate the functional association and synergistic regulatory relationship between *GATA4* and *OCT4*, we employed two complementary bioinformatics tools: GeneMANIA and STRING. By integrating the prediction results from these tools, we identified a series of genes that may have synergistic regulatory effects with *GATA4*, including *SRF*, *NKX2-5*, *TBX5*, *SOX2*, and *NANOG*. Furthermore, based on the enrichment analysis of deep sequencing results, we also predicted several transcription factors that may synergistically interact with *GATA4*, such as *SALL4*, *SRF*, *TBX5*, and *NKX2-5* (Figure 6D,E).

### 3.4. Experimental Validation of Gene Screening

**Figure 7:**
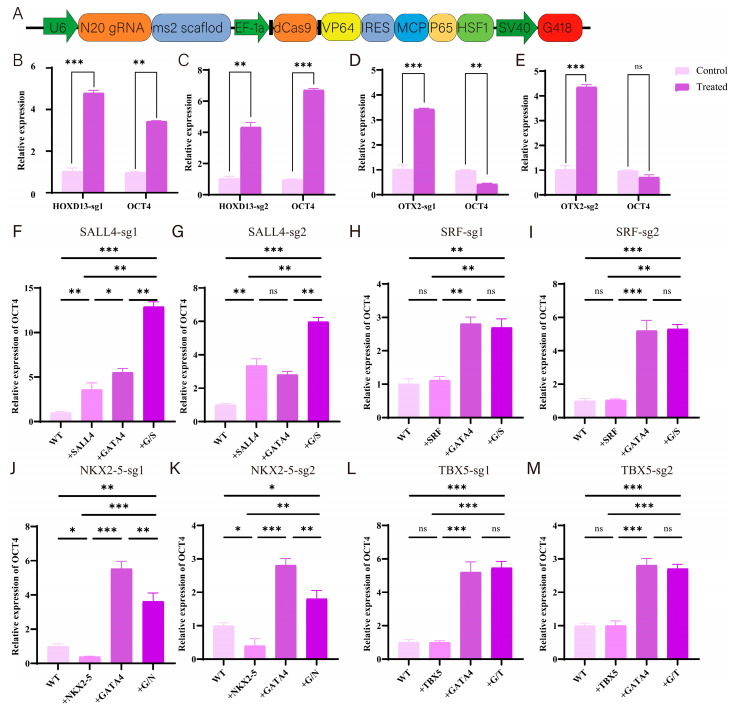
Verifying the regulatory effect of screening transcription factors on *OCT4*. (**A**) A CRISPR activation vector pattern diagram. (**B**–**E**) The regulatory effect of activating plasmids on the expression of *HOXD13*, *OTX2*, and *OCT4*. (**F**–**I**) Regulation of OCT4 expression by transcription factors *SALL4* and *SRF*. (**J**–**M**) Regulation of *OCT4* expression by transcription factors *NKX2-5* and *TBX5*. Note: * in the figure represents the significant difference between the two groups, where *** represents *p* < 0.001, ** represents *p* < 0.01, * represents *p* < 0.05, and ns represents no significant difference.

In order to verify the reliability of library screening, two types of transcription factors were selected for validation in this study, namely *HOXD13* and *OTX2*, which may directly regulate *OCT4* expression, and *SALL4*, *SRF*, *NKX2-5*, and *TBX5*, which may have synergistic regulatory effects with *GATA4*. On the basis of an all-in-one CRISPRa vector, the activation plasmid corresponding to the above transcription factors was constructed (Figure 7A). For each transcription factor to be validated, two sgRNAs were designed.

To investigate the transcriptional regulation of *OCT4*, activation plasmids for the selected transcription factors were electroporated into PK15 cells, with or without *GATA4* overexpression. RT-qPCR analysis confirmed the effective upregulation of target genes post-selection. Among the direct regulators, *HOXD13* activation significantly increased *OCT4* expression, while *OTX2* suppressed it, indicating opposing regulatory roles (Figure 7H,I,L,M). For co-regulators, *SALL4* alone upregulated *OCT4*, and co-activation with *GATA4* led to further enhancement, suggesting a synergistic effect (Figure 7F,G). *SRF* and *TBX5* showed no significant impact on *OCT4* expression under either condition (Figure 7F–M). In addition, the activation of *NKX2-5* alone could downregulate *OCT4* expression, while in GATA4-activated cells, *OCT4* expression was significantly downregulated after *NKX2-5* activation, indicating that *NKX2-5* has an inhibitory effect on *OCT4* expression (Figure 7J,K).

## 4. Discussion

OCT4 is a pivotal regulator of pluripotency during early embryogenesis and in embryonic stem cells [30]. In pigs, as in mice and humans, *OCT4* serves as a hallmark of pluripotency and is frequently used to assess reprogramming efficiency during induced pluripotent stem cell (iPSC) generation [6,7]. However, the regulatory network controlling *OCT4* expression in pigs remains poorly defined. By leveraging a pig’s transcription factor sgRNA library and a dCas9-SAM activation system, we conducted gain-of-function screening to identify transcription factors that regulate *OCT4* expression, both independently and in cooperation with *GATA4*.

To ensure the comprehensiveness and reliability of the genetic screening process, genome-wide sgRNA libraries are typically designed to include multiple sgRNAs per gene, targeting promoter regions or sequences located 50–400 bp upstream of the transcription start site (TSS) to maximize activation efficiency [16]. For each gene, four sgRNAs were carefully designed to target regulatory elements upstream of the TSS, thereby increasing the likelihood of capturing gene-specific activation events. This library design provides a robust platform for the systematic investigation of pig transcription factors and establishes a foundation for exploring gene regulatory networks in pigs.

To identify transcription factors involved in the regulation of *OCT4*, we established a CRISPR/Cas9-engineered reporter cell line in PK15 cells, in which *EGFP* expression is driven by the *OCT4* promoter. This reporter system enables us to visualize *OCT4* promoter activity in real time and facilitates downstream functional screening. To ensure the precise knock-in of the reporter construct, we employed CRISPR/Cas9-mediated targeting of the pigs ROSA26 (pROSA26) locus, which is widely used as a “safe harbor” site due to its evolutionary conservation and stable expression profile [31,32]. To minimize transcriptional interference from the endogenous ROSA26 promoter, the *OCT4* promoter was inserted in the opposite transcriptional orientation, thus improving the fidelity of the reporter readout.

Our screening results revealed several candidate regulators. High-throughput sequencing and MAGeCK analysis identified *MYC*, *PLAG1*, *HOXD13*, *SOX2*, and *PRDM14* as potential activators of OCT4, whereas CDX2, OTX2, THRB, VDR, and OSR2 may act as repressors. Notably, in the presence of *GATA4* overexpression conditions, *SALL4*, *PRDM14*, and *STAT3* were found to enhance *OCT4* expression, suggesting synergistic activity, while *NKX2-5*, *TCF3*, and *CDX2* were associated with repression. These findings are consistent with prior studies reporting the involvement of transcription factors such as *MYC*, *SOX2*, *PRDM14*, *SALL4*, *CDX2*, and *OTX2* in *OCT4* regulation [9,33], thereby validating the robustness of our CRISPRa-based screening strategy.

*GATA4* is a zinc finger transcription factor of the GATA family, which recognizes the conserved GATA motif (A/T-GATA-A/G) in target DNA [12]. All six vertebrate GATA factors share a conserved dual zinc finger DNA-binding domain, composed of N- and C-terminal zinc fingers [34,35]. Previous studies have demonstrated that the N-terminal region of *GATA4* contains two independent activation domains essential for transcriptional activity [36,37]. While the C-terminal region alone is insufficient to activate transcription, it plays a crucial role in post-translational modifications such as acetylation and ubiquitination [38], and is required for cooperative transcriptional activity with other regulators, including *TBX5* and *FOG2* [39]. These structural features underscore the complex regulatory capacity of *GATA4* and its ability to participate in diverse transcriptional programs.

To further explore potential cooperative regulatory mechanisms, we performed bioinformatics analyses using GeneMANIA and STRING databases. These analyses identified several genes, including *SRF*, *NKX2-5*, *TBX5*, *SOX2*, and *NANOG*, as potential co-regulators of *GATA4*. Deep sequencing data indicated that transcription factors such as *SALL4*, *SRF*, *TBX5*, and *NKX2-5* may interact functionally with *GATA4* to influence *OCT4* expression. Subsequent validation experiments showed that *HOXD13* upregulates *OCT4* expression, while *OTX2* suppresses it. Importantly, *SALL4* enhanced *OCT4* expression more significantly in the presence of *GATA4*, suggesting functional synergy. Conversely, the co-expression of *NKX2-5* reduced *OCT4* levels in GATA4-overexpressing cells, although no direct interaction was confirmed. *SRF* and *TBX5* did not exert a detectable regulatory effect on *OCT4* under the conditions tested. These results contribute to further elucidating the transcriptional regulatory mechanism of *GATA4* on *OCT4* in early pig embryos.

This study identified several transcription factors that influence *OCT4* expression in pig cells, with *HOXD13*, *OTX2*, and *SALL4* playing direct or cooperative roles. These findings contribute to a better understanding of the regulatory networks controlling pluripotency in pigs. Future studies should aim to delineate the dynamic interactions of these transcription factors at single-cell resolution across different developmental stages. However, luciferase reporter assays and co-immunoprecipitation experiments should be taken to elucidate the mechanism of transcription factors’ action. Integrating single-cell RNA sequencing, chromatin accessibility profiling, and genome editing technologies will enable a more comprehensive dissection of their regulatory functions. In this study, we only screened in one cell line. As *OCT4* is a multipotent regulatory factor, further functional validation can be conducted in pig-induced pluripotent cells or embryonic stem cells in the future.

## 5. Conclusions

In summary, this study established a robust pig OCT4-EGFP reporter system integrated at the ROSA26 locus and coupled with the dCas9-SAM activation platform to enable high-throughput functional screening. By utilizing a genome-scale sgRNA library targeting pigs’ transcription factors, we systematically identified key regulators of *OCT4* expression, including both activators and repressors, as well as factors that potentially cooperate with *GATA4*. Among the identified candidates, transcription factors such as *HOXD13*, *OTX2*, and *SALL4* were validated for their regulatory roles, revealing both direct and context-dependent effects on *OCT4* expression. These results provide new insights into the transcriptional regulation of *OCT4* and offer a valuable platform for studying pluripotency in pigs.

## Data Availability

The authors declare that all data supporting the findings of this study are available within the paper and its Supplementary Materials.

## Supplementary Materials

The following supporting information can be downloaded at: https://www.mdpi.com/article/10.3390/cells14171330/s1, Table S1: List of Enriched Transcription Factors. Table S2: The primers used in this paper. Table S3: Sequence of tandem tRNA–sgRNA.
