# Supplementary material for: Establishment of a CRISPR/dCas9 Activation Library for Screening Transcription Factors Co-Regulating OCT4 with GATA4 in Pig Cells
Source: Cells. 2025 Aug 28;14(17):1330. doi: 10.3390/cells14171330 (PMC12427770; doi:10.3390/cells14171330)
Supplement: Supplementary file 1 [file cells-14-01330-s001.zip › Supplementary Table S1 List of Enriched Transcription Factors.pdf]

Table List of Enriched Transcription Factors

Activation	Log2_Ratio	Synergistic activation	Log2_Ratio	Repression	Log2_Ratio	Synergistic repression	Log2_Ratio
MYC	12.513728	SALL4	12.513728	CDX2	9.39660478	NKX2-5	9.321928
PLAG1	11.927037	ZBTB22	11.927037	OTX2	8.169925	TCF3	7.658211
HOXD13	10.213728	PRDM14	10.213728	THRB	5.214319	DLX3	6.906891
SOX2	10.121318	STAT3	10.121318	VDR	5.169925	TFAP2E	6.875733
PLAG1	9.914017	TBX5	9.914017	OSR2	5.022368	CDX2	6.475733
SOX6	9.807355	SP2	9.807355	ETV2	4.882643	EN2	6.228819
GATA4	9.625709	TBX10	9.625709	NKX2-5	4.503826	ZBED2	5.951868
IRF5	9.264443	NFIC	9.264443	HEY2	3.898761	BHLHA15	5.69488
EGR2	8.5698556	KLF7	8.5698556	CDC5L	2.623051	ZFP37	5.475733
PRDM15	8.033423	SRF	8.033423	THRB	2.220011	OTX2	5.4411
ZBTB22	7.693487	SP1	7.693487	TCF3	2.107723	ID2	5.337303
SP8	7.033423	ZBTB26	7.033423	HEY2	2.013547	NR2F2	4.882643
NR5A1	6.9068906	YY1	6.9068906	RBAK	1.957885	HIF3A	4.787903
ELK1	6.741467	SKOR1	6.741467	FOXO1	1.877992	CRX	3.921928
KLF4	6.3923174	NR4A1	6.3923174	TAL2	1.645207	NKX2-8	3.321928
NONOG	6.0660892	EGR2	6.0660892	DKK1	1.323529	ARP-1	3.169925
SALL4	5.9307373	BHLHE41	5.9307373	DLX5	1.1764977	EAR-3	2.807355
FOXF2	5.7681843	SNAI2	5.7681843	EOMES	0.9523033	OSR2	2.584963
GATA1	5.7548875	SOX2	5.7548875	HOXA5	0.8039723	HEY2	2.321928
STAT1	5.39231742	KLF4	5.39231742				
FOXA1	5.2159374	TR2	5.2159374				
ATF7	4.86624861	SOX1	4.86624861				
FOXO4	4.7548875	NEUROG3	4.7548875				
PRDM14	4.5844246	USF3	4.5844246				
POU4F3	4.2475265	HOXD9	4.2475265				
SNAI2	4.2479275	SOX11	4.2479275				
PAX7	4.1844246	ST18	4.1844246				
E4F1	3.7004397	PAX4	3.7004397				
IRF1	3.574743	FOXO4	3.574743				
SALL1	3.158305	GATA1	3.158305				
ZSCAN10	12.513728	REST	12.513728				
NOBOX	11.927037	NONOG	11.927037				
ESRRB	10.213728	FOXO1	10.213728				
