# Supplementary material for: Establishment of a CRISPR/dCas9 Activation Library for Screening Transcription Factors Co-Regulating OCT4 with GATA4 in Pig Cells
Source: Cells. 2025 Aug 28;14(17):1330. doi: 10.3390/cells14171330 (PMC12427770; doi:10.3390/cells14171330)
Supplement: Supplementary file 1 [file cells-14-01330-s001.zip › Supplementary Table S2. The primers used in this paper.pdf]

Table S2-1. Primers for amplification of the TK fragment

Primer names	Sequence(5′→3′)
TK-F	AATTGATGGAGACGCGTCGACGTGGCACGTATACTGAGTC ATTA
TK-R	TTCAGATCGAGACGGATCCAGACATGATAAGATACATTGAT GA

Table A2-2. Gene knock-in identification primers

Primer names	Sequence(5′→3′)
OCT4-KI-F	AGGGGACTGCTCGACACCTCTCTCCC
OCT4-KI-R	CCAAACAAAGGCCTGGACGCCTCAG
EGFP-KI-F	GCTGTAGGTATCTCAGTTCGGTGTAGG
EGFP-KI-R	TCTATCGCCTTCTTGACGAGTTCTTCT
3'-KI-F	GCTTCCTGCTCTTCTCTTGTCACTGATTGG
3'-KI-R	GCCTGGTATCTTTATAGTCCTGTCGGGTTTC
5'-KI-F	GTTAAGTCATCAGACCTGGCTTCTCCACTCT
5'-KI-R	AGGACTATACTAGGTGCTTAGGTACAAGACTCAACA
SAM1-F	ATTATCAAGGACAAGGACTTCCTGGACAAT
SAM1-R	AGTAGTTCTTCATCTTCTTCACGACCTCTTC
SAM2-F	GAGAGTGATCCTGGCCGACGCTAATCTG
SAM2-R	CACCATTGTCCACGAGCACGAACTGAGTA
SAM3-F	TTCTGACTGTGAACTCATCGTGAAGGCAAT
SAM3-R	CTGTGACAGCAGGGCACTAAAGTCCATATC

Table S2-3. Q-PCR primers

Primer names	Sequence(5′→3′)
qGATA4-F	ATGAAGCTCCATGGTGTCCC
qGATA4-R	ACTGCTGGAGTTGCTGGAAG
qEGFP-F	TTGTGGCTGTTGTAGTTGTA
qEGFP-R	GCACCATCTTCTTCAAGGAC
qNKX2-5-F	AACGCCTACGGCTATAAC
qNKX2-5-R	CGAAGTTCACGAAGTTGTT
qHOXD13-F	TGAGGCGTACATCTCCAT
qHOXD13-R	CATACTCGTTCTCCAGTTCT
qTBX5-F	GCACAGGATGTCAAGGAT
qTBX5-R	GTTGGATGAGGTGGAGAG
qSRF-F	TACCACCTCCACAATCCA
qSRF-R	TACTCTTCAGCACAGTTCC
qSALL4-F	GAGAGCATCAAGTCCAAGTC
qSALL4-R	CTGGCAGATGAGAAGTTCTT
qOCT4-F	GTGTTCAGCCAAACGACCATC
qOCT4-R	GTCTCTGCCTTGCATATCTCC
qGAPDH-F	CTCAACGGGAAGCTCACTGG
qGAPDH-R	TGATGTCATCATATTTGCAGGTT
