# Supplementary material for: Establishment of a CRISPR/dCas9 Activation Library for Screening Transcription Factors Co-Regulating OCT4 with GATA4 in Pig Cells
Source: Cells. 2025 Aug 28;14(17):1330. doi: 10.3390/cells14171330 (PMC12427770; doi:10.3390/cells14171330)
Supplement: Supplementary file 1 [file cells-14-01330-s001.zip › Supplementary Table S3 Sequence of tandem tRNA–sgRNA.pdf]

Supplementary Table S3 Sequence of tandem tRNA–sgRNA

Name	Sequence（5’->3’）
4×tRNA-sgGATA4	AACAAAGCACCAGTGGTCTAGTGGTAGAATAGTACCCTGCCACGGTACAGACC<br>CGGGTTCGATTCCCGGCTGGTGCACCCAACTTGTGTAGTTCAAGGTTTTAGAGC<br>TAGGCCAACATGAGGATCACCCATGTCTGCAGGGCCTAGCAAGTTAAAATAAG<br>GCTAGTCCGTTATCAACTTGGCCAACATGAGGATCACCCATGTCTGCAGGGCCA<br>AGTGGCACCGAGTCGGTGCAACAAAGCACCAGTGGTCTAGTGGTAGAATAGTA<br>CCCTGCCACGGTACAGACCCGGGTTCGATTCCCGGCTGGTGCATCCTGCTCAAC<br>CCGCGCTTTGTTTTAGAGCTAGGCCAACATGAGGATCACCCATGTCTGCAGGGC<br>CTAGCAAGTTAAAATAAGGCTAGTCCGTTATCAACTTGGCCAACATGAGGATCA<br>CCCATGTCTGCAGGGCCAAGTGGCACCGAGTCGGTGCAACAAAGCACCAGTGG<br>TCTAGTGGTAGAATAGTACCCTGCCACGGTACAGACCCGGGTTCGATTCCCGGC<br>TGGTGCAGCTGTACAACCATGTAATGTGTTTTAGAGCTAGGCCAACATGAGGAT<br>CACCCATGTCTGCAGGGCCTAGCAAGTTAAAATAAGGCTAGTCCGTTATCAACT<br>TGGCCAACATGAGGATCACCCATGTCTGCAGGGCCAAGTGGCACCGAGTCGGT<br>GCAACAAAGCACCAGTGGTCTAGTGGTAGAATAGTACCCTGCCACGGTACAGA<br>CCCGGGTTCGATTCCCGGCTGGTGCATCCACTGGTACACATACCAAGTTTTAGA<br>GCTAGGCCAACATGAGGATCACCCATGTCTGCAGGGCCTAGCAAGTTAAAATA<br>AGGCTAGTCCGTTATCAACTTGGCCAACATGAGGATCACCCATGTCTGCAGGGC<br>CAAGTGGCACCGAGTCGGTGCTTTTTTT
